# Prevalence and risk factors associated with ectoparasite infestation of buffaloes in an Amazonian ecosystem

**DOI:** 10.1186/s13071-018-2917-2

**Published:** 2018-06-04

**Authors:** Helder Ribeiro Batista, Cristiane Sarturi, Felipe Nascimento Stelmachtchuk, Daniel Rocha Oliveira, Adriana Caroprezo Morini, Solange Maria Gennari, Arlei Marcili, Fernanda Aparecida Nieri Bastos, Lauro Euclides Soares Barata, Antonio Humberto Hamad Minervino

**Affiliations:** 1 Laboratório de Sanidade Animal, Universidade Federal do Oeste do Pará, LARSANA/UFOPA, Rua Vera Paz, s/n, CEP 68100-000 Santarém, Pará Brasil; 2 Faculdade de Medicina Veterinária e Zootecnia, Universidade de São Paulo, FMVZ/USP, Av. Prof. Orlando Marques de Paiva 87, Cidade Universitária, São Paulo, SP 05508-270 Brazil; 3 Mestrado em Medicina e Bem estar animal, Universidade Santo Amaro, Av. Prof. Eneas de Siqueira Neto, 340, São Paulo, 04529-300 Brazil; 4 Programa de Pós-Graduação em Recursos Naturais da Amazônia, Universidade Federal do Oeste do Pará, PGRNA/UFOPA, Av. Mendonça Furtado 2240, CEP 68100-000 Santarém, Pará Brasil

**Keywords:** Amazon ecosystem, *Bubalus bubalis*, Buffalo, Ectoparasite, Lice, Tick

## Abstract

**Background:**

The water buffalo (*Bubalus bubalis*) is well adapted in some regions of the Amazon. Of all Brazilian states, Pará contains the largest number of this species, with 510,000 animals, approximately 38% of the Brazilian buffaloes. Despite the socioeconomic importance of bubaline farming in the northern region, little is known about the prevalence of ectoparasites that affect buffalo herds. This study aimed to identify the species of buffalo ectoparasites in the municipality of Santarém, Pará, and to determine possible risk factors related to ectoparasitic infestation. A cross-sectional study was conducted by sampling 60 rural properties and 621 buffaloes for ectoparasites. When present, ectoparasites were collected for subsequent identification.

**Results:**

Of all the animals sampled, 18.5% (115/621) had ectoparasites, 7.8% (49/621) had ticks from the species *Rhipicephalus* (*Boophilus*) *microplus* and *Amblyomma cajennense* (*sensu stricto*), and 11.5% (72/621) had lice from the *Haematopinus tuberculatus* species*.* Six animals presented mixed infestations of ticks and lice. Among the sampled farms, 51.6% (31/60) had at least one animal infested with ectoparasites. The prevalence of ticks and lice on buffaloes was associated with the farm site, with higher prevalence (11.5% ticks, 15.4% lice) in animals at dry land (OR: 16.7 and 5.7 for ticks and lice, respectively) when compared with floodplains (0.5% ticks, 3.4% lice). Buffaloes aged 1 to 12 months had more ticks whereas buffaloes aged 13 to 24 months had more lice (*P* < 0.05).

**Conclusions:**

Buffaloes bred in the municipality of Santarém present different levels of tick and lice infestation according to the direct influence of Amazon ecosystem characteristics. The floodplain environment, widely used for buffalo farming, contributes toward minor ectoparasite infestations in these animals.

## Background

There are 1.78 million buffaloes in Brazil, of which 507,000 (38%) are found in Pará state, northern region of Brazil [1]. In Santarém, and throughout the western region of the state, buffaloes are raised either on dry land (regular farms) or/and inhabit floodplain ecosystems through 6–8 months of the year, when the rivers are drying out and pastures become available. This floodplain production system is extensive and characterized by minimal animal management [2].

Buffaloes are known for being resistant to tick infestations. However, some tick species parasitize buffaloes, although usually at a lower intensity than in cattle [3]. The tick species *Rhipicephalus* (*Boophilus*) *microplus* and *Amblyomma cajennense* complex typically parasitize buffaloes [4–6].

There have been few reports about tick parasitism in buffaloes in Brazil. In the states of São Paulo and Minas Gerais, southeastern region, a natural infestation of *R.* (*B*) *microplus* and *Dermacentor nitens* was reported in buffaloes, the ticks mainly parasitizing the groin and axilla, perineum, udder and other regions where the skin is thinner and the hair shorter [4]. In Rio de Janeiro, also in the southeastern region of Brazil, 82.3% of buffalo herds show tick infestations; nonetheless, other ectoparasites have not been considered in previous studies in this region [7]. *Amblyomma sculptum*, the vector of Brazilian spotted fever, which has low host parasitic specificity, has previously been found in buffaloes [8].

Among the species of ectoparasites that affect buffaloes, *Haematopinus tuberculatus* lice are specific to buffaloes, and thus the main ectoparasite of the species [8, 9]. In Brazil, Silva et al. [10] suggest that *H. tuberculatus* may be involved in transmission of diseases, such as anaplasmosis.

Very little is known about the risk factors related to the presence of ticks or lice in buffaloes, especially in the Amazon region, which presents a unique farming system that strongly utilizes floodplains. This study aimed to identify the species of ectoparasites in buffaloes in the municipality of Santarém, Pará, and to determine the possible risk factors related to parasitic infestation.

## Methods

### Study area

**Fig. 1:**
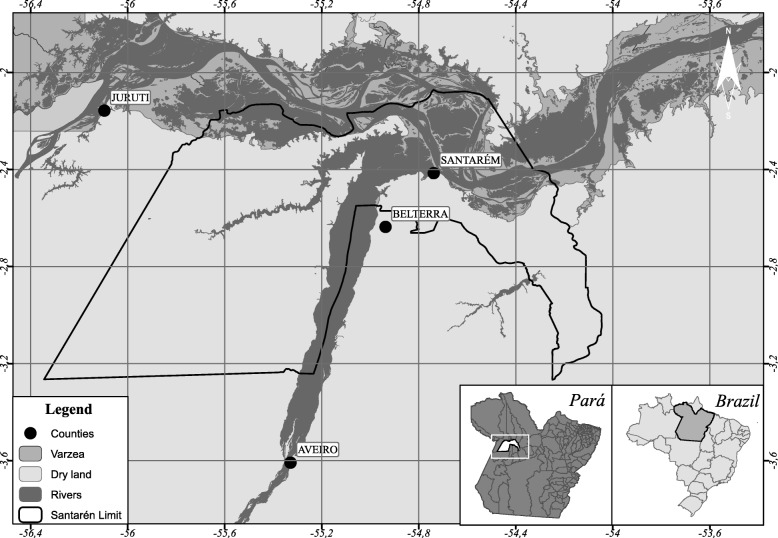
Municipality of Santarém with the dry land and the floodplain areas used for buffalo breeding

This study was carried out in the municipality of Santarém, in the western region of the state of Pará, northern Brazil, and included properties located in dry areas (conventional properties) and in floodplain areas; the latter are extensively found along the banks of the Amazon River and are inundated annually. In addition, during the drought period, these areas provide natural pastures that are used for livestock activities; animals are transported to the floodplains where they remain for up to 8 months of the year, only returning to the dry areas during the peak flood of the Amazon River [11]. The buffalo herd in this municipality consists of approximately 8,364 buffaloes, distributed across 232 properties [1]. Figure 1 presents the studied area.

### Experimental design

A cross-sectional study was carried out with a two-stage sampling procedure. The first stage included sampling of rural properties. Sample size calculation was performed with the software Epi Info (CDC), using the following data: population size: 232 farms; frequency of expected tick infestation (farm-level): 82% [7]; confidence level: 95%; confidence limits: 8.4%. Accordingly, we calculated that 60 farms (clusters) needed to be sampled, representing 25.9% of the total number of properties on which buffaloes were raised in the municipality. To select the farms, a systematic sampling approach was adopted from the list of producers registered at the Agency of Agricultural Defense of the State of Pará in the year of 2015.

The second sampling stage matched the number of animals collected from each rural property, calculated on site considering the total number of buffaloes in the farm according to a statistical formula for disease detection [12], assuming the following values: 95% confidence level; expected prevalence of ticks on buffaloes of 15% [4]; test sensitivity of 95%.

When the farms were visited to collect samples, an epidemiological questionnaire was used to obtain information related to farm location (dry land or floodplains), herd size, type of herd (beef or dairy), type of pasture, type of grazing, presence of mixed overgrowth pasture (presence of undesired plants such as bushes and shrubs in the pasture), pasture resting, control of ectoparasites (if any), and presence of wild animals. Individual details of each animal such as sex, age, breed, type (beef or dairy) and origin (born at farm or bought) were obtained. We also checked if the buffaloes were maintained with other animal species, and if they were transported to floodplains.

### Ectoparasite collection

After appropriate containment, the buffaloes were examined in predefined body areas (neck, ears, back and tip of the tail), and the ectoparasites found were collected from each animal, particularly ticks and lice. The collected ectoparasites were placed into plastic tubes containing absolute alcohol, and were subsequently morphologically identified through specific characteristics [13–15].

### Statistical analysis

Two distinct buffalo conditions were considered for statistical data analyses: presence/absence of ticks and presence/absence of lice. Thus, univariate analysis was performed by Chi-square or Fisher’s exact test (less than five observations in at least one cell in the contingency table) to investigate the association of ticks or lice and possible risk factors obtained through an epidemiological questionnaire. Subsequently, a multivariate model was built by including every hypothesized risk factor which had a *P*-value < 0.200 from the univariate analysis. A logit binary logistic regression with a hierarchic backward stepwise elimination method was used with the lowest risk category was taken as the baseline. Variables were retained if the *P*-value from the logistic regression was < 0.05, otherwise they were removed from the final model. Model validity and reliability was assessed using the Hosmer-Lemeshow goodness-of-fit test [16]. Analyses were performed at two levels: (i) individual animal-level, which considered the prevalence among the entire population sampled, and (ii) herd-level, which evaluated the prevalence across all farms sampled. Each farm was considered a sampling unit, which could be positive (when at least one animal on the property was infested with ticks or lice) or negative (when no animal on the property was infested with ticks or lice).

Statistical analyses were performed using Minitab 17 software (Minitab Inc., State College, USA). Differences were considered significant when *P*-values were ≤ 0.05.

Prevalence and abundance rates, along with mean infestation intensity and mean crowding were calculated, with respective 95% CIs, in accordance with the report by Bush et al. [17], using the Quantitative Parasitology 3.0 software [18]. In this study, prevalence refers to the number of infested buffaloes/number of examined buffaloes × 100. Mean abundance refers to the total number of ticks collected/total number of buffaloes analyzed. Mean intensity refers to the total number of ticks collected/number of infested buffaloes. Mean crowding refers to the degree of species-specific ectoparasite interactions observed on the same host.

## Results

### General characteristics

Sixty farms and 621 buffaloes were sampled. Of these, 34 (56.6%) farms were located on dry land areas, and 26 (43.3%) on floodplain areas. Two farms (3.3%) were considered medium-sized herds (101–500 animals), 24 (40%) were considered small herds (16–100 animals), and 34 (56.6%) were considered micro herds (1–15 animals). Of the 621 animals, 385 (62.0%) were female and 236 (38.0%) were male, 153 (24.6%) were calves (1 to 12 months), 222 (35.7%) were heifers (12 to 24 months) and 246 (39.6%) were adults (over 24 months). The animals were raised for beef (229, 36.9%) and for dairy (392, 63.1%). They belonged to the following breeds: Jafarabadi, 1 (0.16%); Mediterranean, 96 (15.4%); Murrah, 88 (14.1%); and crossbreeds, 436 (70%). From the total sampled 70 (11.2%) were purchased from other farms, and 551 (88.7%) were born at the properties.

Nineteen farms (31%) had cultivated pastures, 36 (60%) farms had native pasture and five (8.33%) farms had mixed pasture (native and cultivated). In terms of grazing management, 42 farms (70%) used the continuous system, 14 (23%) used the alternating system and four (7%) used the rotational system. Mixed overgrowth pastures were present in most of the farms (55/60). Of the farms visited 60% (36/60) controlled ectoparasites (spraying and injectable antiparasitic drugs). Most of the farms (55/60) moved part of their buffalo herd to floodplain areas during a period of the year.

### Prevalence of ectoparasites

Of the 60 sampled properties, 31 (51.6%) contained animals with ectoparasites (lice and ticks); of the 621 buffaloes sampled, 115 (18.5%) had ectoparasites, with 49 animals (7.9%) parasitized by ticks, 72 (11.6%) parasitized by lice (*H. tuberculatus*) and six parasitized by both ticks and lice*.*

A total of 95 ticks were found, including 1 larva, 1 nymph and 93 adults. The nymph and adults were all identified at the species level. The nymph and 48 adults (9 males, 38 females) belonged to *R.* (*B.*) *microplus*, while the other 45 adults (22 males, 23 females) belonged to *Amblyomma cajennense* (*sensu stricto*). The larva was identified as belonging to the genus *Amblyomma.* Five of the tick-parasitized animals presented mixed infestation by the two species of ticks identified. Table 1 shows the number, sex and life-cycle stage of the ectoparasites found on the buffaloes.

**Table 1:** Total number, life-cycle stage and species of ectoparasites identified on buffaloes on 60 farms

Ectoparasite	Larva^a^	Nymph^a^	Adult^a^		Total
			Male	Female	
Ticks					
*Rhipicephalus* (*Boophilus*) *microplus*	–	1/1	9/6	39/19	49/26
*Amblyomma* sp.	1/1	–	22/9	23/13	46/23
Lice					
*Haematopinus tuberculatus*	–	–	320/72		320/72

^a^Total number of ectoparasites found/number of infested buffaloes

Among the two tick species, the most frequently found was *R.* (*B.*) *microplus*, which infested 26 buffaloes, while *A. cajennense* (*sensu stricto*) infested 23 animals. Considering the occurrence of ticks per animals, 23 buffaloes were infested by 1 adult tick, 23 were infested by 2–4 adult ticks, and 3 animals were infested by 5 or more ticks. Regarding the *R.* (*B.*) *microplus* species, 13 buffaloes were infected by 1 tick, and 12 buffaloes were infected by 2–4 ticks. Regarding the *A. cajennense* (*sensu stricto*), 10 buffaloes were infected by 1 tick, 11 animals were infected by 2 to 4 ticks, and 2 animals were infected by 5 ticks. Table 2 presents the prevalence, mean intensity, mean abundance, mean crowding, and their respective 95% CI values of tick infestation on the parasitized buffaloes.

**Table 2:** Prevalence, intensity, abundance and crowding of tick infested buffaloes in Santarém, Pará, Brazil

Tick	Prevalence (%)^a^ (95% CI)	Mean intensity^b^ (95% CI)	Mean abundance^c^ (95% CI)	Mean crowding^d^ (95% CI)
*Rhipicephalus* (*Boophilus*) *microplus*	4.03 (2.62–5.89)	2.00 (1.56–2.40)	0.08 (0.05−0.11)	2.60 (2.14–3.07)
*Amblyomma cajennense* (*sensu stricto*)	3.70 (2.36–5.51)	2.00 (1.52–2.57)	0.07 (0.05–0.11)	2.74 (2.06–3.59)

^a^The number of infested buffaloes/number of examined buffaloes × 100

^b^Total number of collected ticks/number of infested buffaloes

^c^Total number of collected ticks/total number of analyzed buffaloes

^d^The degree of interaction of a parasite with individuals of the same species in the same host

*Abbreviation*: CI, confidence interval

We do not present information on the number of lice per animal as, depending on the collection conditions, the number of individuals collected may differ from the total number of ectoparasites present on the animal. However, all collected lice were of the *H. tuberculatus* species, and were at the adult stage of their life-cycle.

### Analysis of risk factors for the presence of ticks and lice

The prevalence of ticks and lice on farms (herd-level) was 31.6% (19/60) and 33.3% (20/60), respectively. Table 3 shows the results of the univariate analysis of risk factors related to the presence of ticks and lice on the farms. Regarding tick infestation at the herd-level, the logistic regression model resulted in the farm site detected as the only significative risk factor, with farms located on dry land being more infested by ticks (OR = 25, 95% CI: 3.0–205.9, *P* = 0.001) than farms on the floodplain. The logistic regression for lice infestation at the herd-level (data not shown) had a significant result (*P* = 0.038) for the Hosmer-Lemeshow goodness-of-fit test, thus the model does not fit with the data and the predicted probabilities deviate from the observed probabilities in a way that the binomial distribution does not predict.

**Table 3:** Univariate analysis of risk factors related to the presence of ticks and lice at the farm-level in the municipality of Santarém, Brazil

Risk factor	Ticks			Lice		
	Positive/total (%)	*P* ^a^	OR (95% CI)	Positive/total (%)	*P* ^a^	OR (95% CI)
Farm location		< 0.001	24.00 (2.91−198.10)		0.002	7.66 (1.93−30.43)
Dry land	17/34 (50.0)			17/34 (50.0)		
Floodplain	1/26 (3.85)			3/26 (11.5)		
Herd size		na	na		na	na
Medium	0/2 (0.0)			1/2 (50.0)		
Micro	6/34 (17.6)			6/34 (17.6)		
Small	12/24 (54.1)			13/24 (54.1)		
Animals go to floodplain area		0.003	0.25 (0.03−1.64)		0.741	0.72 (0.11−4.76)
Yes	15/55 (27.2)			18/55 (32.7)		
No	3/5 (60.0)			2/5 (40.0)		
Type of pasture		0.019	^b^		0.066	na
Cultivated	9/19 (47.3)			6/19 (31.5)		
Mixed	3/5 (60.0)			4/5 (80.0)		
Native	6/36 (16.6)			10/36 (27.7)		
Type of grazing		0.001	^c^		0.097	na
Alternating	9/14 (64.2)			8/14 (57.1)		
Continuous	5/42 (11.9)			11/42 (26.1)		
Rotational	4/4 (100.0)			1/4 (25.0)		
Mixed overgrowth pastures		0.126	0.27 (0.04−1.79)		0.509	2.11 (0.22−20.26)
Yes	15/55 (27.7)			19/55 (34.5)		
No	3/5 (60.0)			1/5 (20.0)		
Control ectoparasites		0.066	2.41 (0.73−7.96)		< 0.001	11.00 (2.24−53.86)
Yes	14/36 (38.8)			18/36 (50.0)		
No	4/24 (16.6)			2/24 (8.3)		
Presence of wild animals		0.907	0.91 (0.20−4.12)		0.443	0.57 (0.13−2.41)
Yes	15/51 (31.3)			16/51 (31.3)		
No	3/9 (33.3)			4/9 (44.4)		

^a^Calculated using Chi-square or Fisher's tests

^b^Type of pasture: cultivated *vs* mixed (OR: 0.60, *P* = 1.00); cultivated *vs* native (OR: 4.50, *P* = 0.015); mixed *vs* native (OR: 7.50, *P =* 0.06)

^c^Type of grazing: alternating *vs* continuous (OR: 13.32, *P* < 0.0001); alternating *vs* rotational (OR: 0.19, *P* = 0.278); continuous *vs* rotational (OR: 0.02, *P* = 0.0008)

*Notes*: A farm was considered positive when at least 1 of the examined animals was infested with lice or ticks. Herd size: micro (1−15 animals); small (16−100 animals); medium (101−500 animals). Mixed overgrowth is the presence of undesired plants such as bushes and shrubs in the pasture

*Abbreviations*: OR, odds ratio; CI, confidence interval; na, not available.

Of the 621 buffaloes evaluated, 49 (7.8%) were infested by ticks and 72 (11.5%) were infested by lice. Table 4 shows the risk factors linked to the presence of ectoparasites in the buffaloes (individual animal-level). Murrah (ticks: 6/88, 6.8%; lice: 12/88, 13.6%), Mediterranean (ticks: 4/96, 4.2%; lice: 1/96, 1.0%) and crossbreed animals (ticks: 39/436, 8.9%; lice: 59/436, 13.5%) were infested by ticks and lice. The single Jafarabadi buffalo examined had no ticks or lice. There was no association between buffalo breed and the prevalence of ticks or lice.

**Table 4:** Univariate analysis of risk factors related to infestations of buffaloes by ticks and lice (individual animal-level) in the municipality of Santarém, Amazon region, Brazil

Risk factor	Ticks			Lice		
	Positive/total (%)	*P* ^a^	OR (95% CI)	Positive/total (%)	*P* ^a^	OR (95% CI)
Sex		0.908	1.03 (0.56−1.88)		0.260	0.74 (0.43−1.20)
Male	19/236 (8.0)			49/385 (12.7)		
Female	30/385 (7.7)			23/236 (9.7)		
Age (months)		< 0.001	^b^		0.002	^d^
1–12	29/153 (18.9)			10/153 (6.5)		
13–24	10/222 (4.5)			39/222 (17.5)		
> 24	10/246 (4.0)			23/246 (9.3)		
Type		0.209	1.50 (0.79−2.86)		0.149	1.48 (0.86−2.53)
Dairy	35/392 (8.9)			21/229 (9.1)		
Beef	14/229 (6.1)			51/392 (13.0)		
Herd size		0.006	^c^		0.025	^e^
Micro	12/218 (5.5)			17/218 (7.8)		
Small	37/347 (10.6)			51/347 (14.7)		
Medium	0/56 (0.0)			4/56 (7.1)		
Farm site		< 0.001	25.81 (3.53−188.50)		< 0.001	5.07 (2.28−11.29)
Dry land	48/420 (11.5)			65/420 (15.4)		
Floodplain	1/201 (0.5)			7/201 (3.4)		
Origin		0.102	1.88 (0.87−4.07)		0.020	
Bought	9/70 (12.8)			14/70 (20.0)		
Born on farm	40/551 (7.2)			58/551 (10.5)		
Maintained with other animals		0.010	0.46 (0.25−0.84)		0.249	1.38 (0.79–2.40)
Yes	25/420 (5.9)			53/420 (12.6)		
No	24/201 (11.9)			19/201 (9.4)		

^a^Calculated using Chi-square or Fisher's tests

^b^Age (months): 1–12 *vs* 13–24 months (OR: 4.96, *P* = 0.001); 1–12 *vs* > 24 months (OR: 5.52, *P* = 0.001); 13−24 *vs* > 24 months (OR: 1.11, *P* = 0.814)

^c^Herd size: micro *vs* small (OR: 0.49, *P* = 0.033); micro *vs* medium (OR: 6.84, *P* = 0.134); small *vs* medium (OR: 13.65, *P* = 0.005)

^d^Age (months): 1–12 *vs* 13–24 months (OR: 0.33, *P* = 0.002); 1–12 *vs* > 24 months (OR: 0.68, *P* = 0.321); 13−24 *vs* > 24 months (OR: 2.07, *P* = 0.009)

^e^Herd size: micro *vs* small (OR: 0.49, *P* = 0.014); micro *vs* medium (OR: 1.10, *P* = 1.000); small *vs* medium (OR: 2.24, *P* = 0.146)

*Abbreviations*: OR, odds ratio; CI, confidence interval

*Note*: Herd size: micro (1−15 animals); small (16−100 animals); medium (101−500 animals)

The binary logistic regression model of tick and lice infestation at the individual animal-level is presented in Table 5. The factors age, farm site and origin of animal were associated with a higher infestation of ticks in the multivariate analysis. Animals raised on dry land are 16.7 times more likely to be infested by ticks than buffalo raised at floodplains (95% CI: 2.3–123.3; *P* = 0.006). Animals aged 1-12 months were six times more likely to be infested with ticks than those aged > 24 months (95% CI: 2.5–14.5; *P =* 0.0001) and buffaloes bought from other farms were more likely to be infested by ticks than animals born on farm (OR: 3.6; 95% CI: 1.4–9.2; *P* = 0.007). For lice, age and farm site were retained in the final model and related with a higher infestation. Buffalo aged 13–24 months had more lice than young animals (1–12 m) (OR: 3.8; 95% CI: 1.8–8.0; *P* = 0.001). Similarly as tick infestation, buffalo raised on dry land are more likely to have lice than animals at floodplains (OR: 5.7; 95% CI: 2.6–12.8; *P* = 0.001).

**Table 5:** Logit binary logistic regression of tick and lice infestation in buffaloes in the municipality of Santarém, Amazon region, Brazil

Variable^a^	Ticks			Lice		
	OR	95% CI	*P* ^b^	OR	95% CI	*P* ^b^
Age (months)						
1 to 12	5.99	2.48–14.51	0.001	Baseline		
13 to 24	1.05	0.42–2.64	0.922	3.83	1.83–8.01	0.001
> 24	Baseline			2.07	0.95–4.53	0.069
Farm site						
Dry land	16.68	2.26–123.27	0.006	5.72	2.55–12.83	0.001
Floodplain	Baseline			Baseline		
Origin						
Bought	3.61	1.42–9.15	0.007	–	–	
Born on farm	Baseline			–	–	

^a^Only factors that remained significantly associated in the final model (*P* < 0.05) are included

^b^Logit binary regression analysis with Backward stepwise elimination in a hierarchic model. Hosmer-Lemeshow goodness-of-fit test: *P* = 0.233 (ticks), *P* = 0.480 (lice)

*Abbreviations*: OR, odds ratio; CI, confidence interval

## Discussion

In the present study, 7.9% of all examined animals had ticks, which is lower than the prevalence rates found in Pakistan (52.5% [19]), Cuba (27.7%) [20] and India (56.7%) [21]. The prevalence of ticks at the herd-level was 31.6%, which was less than the 82.3% prevalence rate found in Rio de Janeiro farms [7]. Buffaloes raised in the Santarém region were infested by two tick species, *R.* (*B.*) *microplus* and *A. cajennense* (*sensu stricto*), both of which have already been identified as parasites typically found in buffaloes [8, 22].

Of the 60 sampled farms, 33.3% harbored animals infested by *H. tuberculatus* lice, indicative of the prominence of this parasite in bubaline species and their presence in the farms in this region. Regarding the prevalence of infested animals with lice our prevalence rate of 11.6 % was close to the infestations rates found in Italy [23] and Pakistan [24].

Of the two tick species identified, the *R.* (*B.*) *microplus* and *A. cajennense* (*sensu stricto*) presented similar prevalence, intensity, abundance and mean crowding. In Minas Gerais State, Brazil, the prevalence of *R.* (*B.*) *microplus* ticks in buffaloes ranges between 15.4–38.5% throughout the year [4]. Considering the lack of data from buffaloes, our results from tick abundancy, intensity and crowding are hard to discuss, and more studies need to be done with evaluation of infestation in different seasons. It is important for report that the samples were obtained during the dry season, the period when it is possible to raise buffalos in floodplain areas since during the wet season the fields are flooded [2].

The univariate analysis at the herd-level demonstrated an association of tick infestation with different risk factors (type of pasture, type of grazing, etc.), but only the farm site was retained in the final logistic regression model, indicating that other risk factors were associated with the main effect of farm site. Farms on dry land had animals with more ticks and this difference could be explained by the biological cycle of ticks, which may be interrupted when flooding occurs between February and June. The periodic flooding of grazing area and the constant movement of animals to reach newly unflooded areas during the ebb period (e.g. the period when the river is drying), are both factors that may reduce the tick population in the environment and animal infestation [25].

At the individual animal-level, the multivariate analysis of tick infestations included in the final model the follow risk factors: farm site, age and origin of animals. Young buffaloes were more susceptible to tick infestation, consistent with previous literature data [19]. In our study, animals aged between 1 and 12 months were six times more likely to be infested with ticks than those aged > 24 months. Sex, breed and aptitude were not associated with tick infestation in buffaloes. Similarly to the herd-level analysis, the animals raised in floodplains had lower tick infestation. Previous research in India demonstrated that animals brought for grazing on pasture lowlands had lower mature tick populations when compared with stall-fed buffaloes on dairy farms and this occurred supposedly because of predation of ticks by birds in the field [26].

Considering the origin of animals (born on property or bought from other farms) the buffaloes that were bought from other farms were more likely to be infested by ticks. This was probably due to the source of animals, usually bought from dry land for the purpose of reproduction [2].

The univariate analysis of lice infestation at the herd-level indicated association with several risk factors (age, herd size, origin), but the multivariate analysis for lice infestation at the herd-level presented a model that does not fit to the data, probably due to a limited amount of data (*n* = 60 farms) and similarity of results; these data are, therefore, not shown.

The multivariate analysis of lice infestations at the individual animal-level included only age and farm site in the final model. Dry land animals were 5.7 times more likely to be infested with lice than floodplain animals, which might be explained by the biological cycle of *H. tuberculatus*, which is completed within the host body. Moreover, maintenance of the parasite on the body of floodplain animals may be impaired by the daily periods spent in the water, searching for food. The parasites are found in greater concentrations around the ears, base of horns, side of the neck, around the scrotum or udder, and especially at the tip of the tail [15, 27]. Some of these areas would be submerged during water grazing or movement of the buffaloes in floodplain areas.

In this study, animals aged over 12 months had a greater prevalence of lice than young animals (aged 1-12 months). Studies indicate that young buffaloes are more susceptible to infestation by *H. tuberculatus* [28]. Our results may be related to the grazing habits of buffaloes in floodplain areas, where part of the body remains submerged during grazing and movement across pastures. Given that younger animals are smaller, a more substantial proportion of the body would be submerged while accompanying adult animals, possibly explaining the lower prevalence of lice in younger animals observed in our study.

Despite presenting significant results of the univariate analysis the risk factors herd size and origin of animal were not significant in the multivariate analysis and removed by backward stepwise elimination from the final regression model. For lice, only age and farm site were associated with the parasite infestation.

The ticks and the lice founded in buffaloes have been previously related as vectors of important pathogens such *Babesia* sp. and *Anaplasma marginale* [10, 29, 30], but limited information is available regarding piroplasmosis in cattle from Santarém and nearby municipalities. Thus, further studies are required to investigate whether buffaloes act as a significant reservoir of tick species and also the role of buffaloes in the epidemiology of piroplasmosis in ruminants in the region.

Buffaloes bred in the municipality of Santarém presented different levels of infestation by ticks and lice, which was mainly influenced by the unique characteristics of the Amazon ecosystem. The floodplain environment, widely used for buffalo farming, contributes to the minor infestation rates by ectoparasites in these animals.

## Conclusions

Buffaloes bred in the municipality of Santarém present different levels of tick and lice infestation according to the direct influence of Amazon ecosystem characteristics. The floodplain environment, widely used for buffalo farming, contributes toward minor ectoparasite infestations in these animals.

## Abbreviation

CI: confidence interval
